# Nicotinamide inhibits melanoma in vitro and in vivo

**DOI:** 10.1186/s13046-020-01719-3

**Published:** 2020-10-07

**Authors:** Francesca Scatozza, Federica Moschella, Daniela D’Arcangelo, Stefania Rossi, Claudio Tabolacci, Claudia Giampietri, Enrico Proietti, Francesco Facchiano, Antonio Facchiano

**Affiliations:** 1 IDI-IRCCS, Istituto Dermopatico dell’Immacolata, Rome, 00167 Italy; 2 Department of Oncology and Molecular Medicine, Istituto Superiore di Sanità, Rome, 00161 Italy; 3 Unit of Human Anatomy, Department of Anatomy, Histology, Forensic Medicine and Orthopedics, Sapienza University, 00161 Rome, Italy

**Keywords:** Melanoma, Nicotinamide, Vitamin B3, Melanoma mouse-animal model, Metabolism, Sirtuin 2, HCAR2, HCAR3, Dietary intake

## Abstract

**Background:**

Even though new therapies are available against melanoma, novel approaches are needed to overcome resistance and high-toxicity issues. In the present study the anti-melanoma activity of Nicotinamide (NAM), the amide form of Niacin, was assessed in vitro and in vivo.

**Methods:**

Human (A375, SK-MEL-28) and mouse (B16-F10) melanoma cell lines were used for in vitro investigations. Viability, cell-death, cell-cycle distribution, apoptosis, Nicotinamide Adenine Dinucleotide^+^ (NAD^+^), Adenosine Triphosphate (ATP), and Reactive Oxygen Species (ROS) levels were measured after NAM treatment. NAM anti-SIRT2 activity was tested in vitro; SIRT2 expression level was investigated by in silico transcriptomic analyses. Melanoma growth in vivo was measured in thirty-five C57BL/6 mice injected subcutaneously with B16-F10 melanoma cells and treated intraperitoneally with NAM. Interferon (IFN)-γ-secreting murine cells were counted with ELISPOT assay. Cytokine/chemokine plasmatic levels were measured by xMAP technology. Niacin receptors expression in human melanoma samples was also investigated by in silico transcriptomic analyses.

**Results:**

NAM reduced up to 90% melanoma cell number and induced: i) accumulation in G1-phase (40% increase), ii) reduction in S- and G2-phase (about 50% decrease), iii) a 10-fold increase of cell-death and 2.5-fold increase of apoptosis in sub-G1 phase, iv) a significant increase of NAD^+^, ATP, and ROS levels, v) a strong inhibition of SIRT2 activity in vitro. NAM significantly delayed tumor growth in vivo (*p* ≤ 0.0005) and improved survival of melanoma-bearing mice (*p* ≤ 0.0001). About 3-fold increase (*p* ≤ 0.05) of Interferon-gamma (IFN-γ) producing cells was observed in NAM treated mice. The plasmatic expression levels of 6 cytokines (namely: Interleukin 5 (IL-5), Eotaxin, Interleukin 12 (p40) (IL12(p40)), Interleukin 3 (IL-3), Interleukin 10 (IL-10) and Regulated on Activation Normal T Expressed and Secreted (RANTES) were significantly changed in the blood of NAM treated mice, suggesting a key role of the immune response. The observed inhibitory effect of NAM on SIRT2 enzymatic activity confirmed previous evidence; we show here that SIRT2 expression is significantly increased in melanoma and inversely related to melanoma-patients survival. Finally, we show for the first time that the expression levels of Niacin receptors HCAR2 and HCAR3 is almost abolished in human melanoma samples.

**Conclusion:**

NAM shows a relevant anti-melanoma activity in vitro and in vivo and is a suitable candidate for further clinical investigations.

## Background

Nicotinamide (NAM) is the amide form of niacin (vitamin B3 or vitamin PP). It is a precursor of nicotinamide-adenine dinucleotide NAD^+^ [1] and is known to play an essential role in energy metabolism and to act in several tissues including skin [2], nervous system [3, 4], and muscles [5]. Its metabolic pathway is related to tryptophan metabolism [6]. NAM is a key player in the nervous system as well as in skin physiology and immune response control [7, 8]. A severe reduction of NAM levels leads to serious pathologic conditions such as pellagra, characterized by the 3Ds symptoms, namely: Dementia, Diarrhea, and Dermatitis. If untreated, pellagra may be fatal. NAM has been recently suggested as a possible candidate to treat preeclampsia and to improve fetal growth [9]; it was shown to reduce transepidermal water loss [10] and is currently recommended to reduce the incidence of non-melanoma skin cancers in high risk individuals [11, 12]. NAM is used to treat acne vulgaris, melasma, atopic dermatitis, and rosacea [13, 14]. The possible side effects and consequences of excessive NAM intake include increased risk of diabetes, Parkinson’s disease, and liver damage [14]; nevertheless, NAM is generally considered a drug with a safe toxicity profile at daily doses of up to 3 g. The role played by NAM and vitamin B3 in cancer control and cancer metabolism is currently under intense investigation [15–17] and several clinical trials assessing their effects in human cancers are ongoing [18]. NAM anticancer action is likely related to the ability to improve repair of the UV-induced DNA damage and to the key role in cellular energy metabolism [19]. Its chemo-preventive role in non-melanoma skin cancer is well known [20, 21] and is related, at least in part, to a direct anti-inflammatory activity [22]. At the present time, a role of NAM in melanoma treatment or prevention has been proposed but not demonstrated [22, 23]. Itzhaki and collaborators [24] showed that NAM inhibits vasculogenic mimicry, an alternative vascularization pathway observed in highly aggressive melanoma, by using ex vivo derived 3D primary melanoma cell cultures, and showed in vitro effects of NAM on proliferation, invasion, and cell cycle profile of melanoma cells. An additional study showed effects on melanoma cell migration and metastasis in B16-F1 cells both in vitro and in vivo models, but no impact was reported on the tumor growth rate in transplanted mice at the doses analyzed [25]. NAM intracellular levels are controlled by Sirtuins, a highly conserved family of class III deacetylase proteins. Sirtuins catalyze a reaction where NAD^+^ is used to remove an acetyl group from a lysine residue and release NAM and acetyl-ribose as end products. Additional studies have shown the anti-melanoma action of sirtuins inhibitors, [25–27], indicating that inhibiting sirtuins may represent an effective way to control melanoma growth [28]. As recently pointed out, the role played by NAM in melanoma needs further investigation [29]. NAM is a central player in controlling energy metabolism. It is a NAD^+^ precursor; its effects have been investigated in energy and Reactive Oxygen Species (ROS) production [30], as well as in inflammation control [31, 32]. Given its central role in controlling energy production and the activity of many enzymes, NAM has been proposed in the treatment of several clinical conditions, including chemoprevention of non-melanoma skin cancer [12, 21, 33] and chemoprevention of lung cancer [34]. We have recently identified novel molecules showing anti-melanoma activity and characterized their mechanism of action [35–39]. In the present study, we aimed at investigating NAM antitumor mechanisms of action and addressed its effects in melanoma models both in vitro and in vivo. In melanoma cells, we show that NAM induces a significant increase of NAD^+^, ATP, and ROS levels, a strong effect on cell cycle phases distribution, and a significant anti-melanoma effect both in vitro and in vivo.

## Materials and methods

### Cell culture

Human melanoma cell lines SK-MEL-28 and A375 were purchased from the American Type Culture Collection (ATCC, Manassas, VA). SK-MEL-28 and A375 were grown respectively in Minimum Essential medium Eagle (MEM; Hyclone, South Logan, UT, USA) and Dulbecco’s modified Eagle’s medium (DMEM; Hyclone) supplemented with 10% fetal bovine serum (FBS; Hyclone), 2 mM L-glutamine, and 100 mM penicillin/streptomycin (Invitrogen, Carlsbad, CA, USA), as previously reported [38]. B16-F10 mouse melanoma cells from ATCC were grown in DMEM containing 10% FBS (Hyclone), 2 mM L-glutamine and 100 mM penicillin/streptomycin (Invitrogen), at 37 °C with 5% CO_2_.

### Cell proliferation

NAM effect on cell number was measured by cell counting. SK-MEL-28 were plated at 1 × 10^5^ cell/plate and A375 and B16-F10 cells were plated at 8 × 10^4^ cell/plate in p35 Petri dishes at time 0. Cells were then starved for 18 h in serum-free medium and the next day treated with NAM 1 , 20 , and 50 mM in complete fresh medium containing FBS 10%. Cells were then harvested with 0.25% trypsin, 2.21 mM EDTA, 1x sodium bicarbonate (Corning, Manassas, VA, USA) and counted at different times (24 and 48 h). NAM was from DSM Nutritional products Ltd. (CH-4002 Basel, Switzerland) (Niacinamide PC, code 5006066). Trypan blue exclusion was used to discriminate live from dead cells.

### Cell-cycle investigation by FACS analysis

A375 cells were plated at 8 × 10^4^ cell/plate in p35 Petri dishes at time 0 and then were starved for 18 h in serum-free medium. The next day, cells treated with NAM (10, 20 and 50 mM) in complete fresh medium for 24 h were harvested by 0.25% trypsin incubation, washed with cold phosphate-buffered saline (PBS) and fixed in 70% ethanol. Cells were then incubated with 1 μg/ml propidium iodide (Sigma) for 3 h at room temperature and then examined by using a BD Accuri C6 Plus Flow Cytometer (BD Biosciences, USA). Data were analyzed by FlowJo software by BD Biosciences.

### Evaluation of energy-related metabolism

The total soluble NAD^+^ level was measured using the NAD^+^/NADH assay kit based on the enzymatic cycling reaction (BioVision, Milpitas, California). SK-MEL-28 were plated at 1 × 10^5^ cells/plate and A375 and B16-F10 cells were plated at 8 × 10^4^ cell/plate in p35 Petri dishes at time 0 and then starved for 18 h in serum-free medium. After 6 h NAM treatment (1, 20, and 50 mM) in the presence of 10% FBS, cells were washed with PBS, harvested with 0.25% trypsin and counted. According to the manufacturer’s instructions, the cell lysate absorbance was measured at 450 nm after 6 h and NAD^+^ concentration was expressed in pmol/μl. Intracellular ATP content was measured by using the ATP Colorimetric/Fluorometric Assay Kit (BioVision). SK-MEL-28 and A375 cells were treated with NAM (1, 20, and 50 mM) for 6 h. Cells were then washed with PBS, harvested and ATP was measured following the manufacturer instructions upon cell lysis. This assay reports the ATP concentration expressed as nmol/10^6^ cells.

ROS level was measured using the 2′,7′-dichlorodihydrofluorescein diacetate (DCFDA)-Cellular Reactive Oxygen Species (ROS) Detection Assay Kit (AbCam ab 113,851). Cells were incubated with 25 μM DCFDA for 45 min at 37 °C and then treated with 50 mM NAM in complete fresh medium for 6 h. Tert-Butyl Hydrogen Peroxide (TBHP) solution was used as a positive control for ROS production. The fluorescence intensity of control and treated wells was measured with the Ensight instrument (Perkin Elmer, Inc. Beaconsfield UK) at Ex = 485 nm and Em = 535 nm, according to the manufacturer’s instructions.

### SIRT2 activity assay in vitro

NAM (from 0.01 mM up to 20 mM) was used to test the effect on SIRT2 activity by using the Sirt2 Inhibitor Screening Assay Kit (Fluorometric) (BioVision). Five μl of purified SIRT2 enzyme was added to each well and then incubated with 45 μl of NAM increasing concentrations and incubated for 5 min at 37 °C. Forty μl of the substrate solution was then added to each well, mixed, and incubated for 60 min at 37 °C. The fluorescence intensity of each sample was measured before (R0) and after (R1) Developer addition, according to the manufacturer’s instructions. The Ensight instrument (Perkin Elmer, Inc.) was used, at Ex = 395 nm and Em = 541 nm. Doses expressed in mM concentration were log-transformed; data were then fitted with the non-linear regression equation and the EC value was calculated by GraphPad Prism 5 (GraphPad Software Inc.).

### Sirtuins transcriptomic analyses and survival analysis

SIRT2 expression levels were investigated in NCI-60 cancer cell lines, a collection of 60 human cell lines from 9 cancer types, reported in the expression array GDS1761 within the GEO database [40]. SIRT2 expression levels in human specimens were from 211 normal skin individuals (controls) and 148 melanoma patients. More in detail, expression values were taken from GENT2 database available at http://gent2.appex.kr/gent2/ [41]. Normal skin data were from 10 datasets, namely: GSE13355, GSE14905, GSE15605, GSE16161, GSE30355, GSE39612, GSE42109, GSE46239, GSE7307, GSE7553); metastatic melanoma data were from 3 experiments, namely GSE77553, GSE19234, GSE22968.

Expression levels of SIRT2 in melanoma and vs control normal skin were also obtained taken from “Pan-Cancer Analysis of Whole Genomes” reported by the Expression Atlas at EBI [42].

Survival analysis in melanoma patients was related to SIRT1, SIRT2, SIRT3, SIRT4, SIRT5, SIRT6, and SIRT7 expression levels reported by GEPIA2 database [43] available at http://gepia2.cancer-pku.cn/#survival. Survival analyses were carried out on quartile distribution with the cutoff for high expression set at 70% and the cutoff for low expression set at 25%, using the skin cancer melanoma (SKCM) database.

### Mouse melanoma model in vivo

The metastatic cell model B16-F10 was used as in vivo model of melanoma. Cells were expanded for a few passages and aliquots were frozen in liquid nitrogen. From each aliquot of parental stock, a second batch of aliquoted and frozen cells was generated. They were thawed before the tumor was implanted. Cells were routinely examined for Mycoplasma contamination. Thirty-five 8-week-old C57BL/6 mice were obtained from Charles River Laboratories (Calco, Italy). Mice were housed in a pathogen-free facility of the Istituto Superiore di Sanità (Rome, Italy) under light- and temperature-controlled conditions and treated in accordance with the European Community guidelines. Experiments were approved by the ISS Review Board (Protocol number 986/SSA/13). Tumors were obtained by subcutaneous (s.c.) injection of 3 × 10^5^ B16-F10 cells. NAM was freshly dissolved in saline solution and administered at 1000 - 1500 - 1800 mg/Kg doses by daily intraperitoneal (i.p.) injection either with a 5 days per week or a 7 days per week schedule. Control mice received the i.p. injection of saline solution.

Tumor growth was monitored twice a week by measuring the size of the tumor with a digital caliper reported as mean tumor diameter. Toxicity was evaluated by mice examination and body weight assessment. Mice survival was analyzed by the Kaplan-Meier method, using as endpoint the day when the tumors reached a mean diameter of 12 mm or the day of euthanasia. Mice were sacrificed if the tumor was necrotic, if the tumor diameter exceeded 16 mm or if the mice showed any sign of distress or weight loss.

### Interferon-gamma ELISPOT

Fourteen days following tumor injection into C57BL/6 mice, blood samples were collected from the retro-orbital plexus from individual mice in K3EDTA anticoagulant-coated tubes (MINIPLAST, LP Italiana SPA). Plasma was separated from cell fractions by low-speed centrifugation and stored at − 80 °C for cytokine expression analysis (see below).

A cytokine enzyme-linked immunospot (ELISPOT) assay designed for measuring the number of interferon (IFN)-γ-secreting cells in PBMCs in response to specific irradiated (20 Gy) B16-F10 tumor cells was performed. Briefly, nitrocellulose-bottomed 96-well plates (MultiScreenTM-IP, Millipore, Bedford, MA) were coated with anti-mouse IFN-γ capture monoclonal antibody (mAb) (Mabtech, Nacka, Sweden), blood leukocytes (1 × 10^5^) were incubated with or without 2 × 10^5^ irradiated tumor cells in triplicate wells. Positive control included incubation of cells with Concanavalin A (Sigma Aldrich, USA). After 24 h at 37 °C, the plate was washed and then incubated with biotinylated anti-mouse IFN-γ mAb (Mabtech), streptavidin Alkaline Phosphatase (ALP) (Mabtech), and ALP-substrate (BCIP/NBT) (Sigma-Aldrich) according to manufacturer’s instructions. The reaction was terminated by washing with tap water upon the appearance of dark spots which were then counted using the 4-Plate Elispot Reader V2.1 (A.EL.VIS, Hannover, Germany).

### Cytokines expression in mice plasma

Fourteen days after tumor injection, plasma samples were obtained by individual mice and stored at − 80 °C. Conditioned media from treated cells were collected, centrifuged and stored at − 80 °C until use. Cytokines levels were measured through xMAP multiplex technology by a Mouse Magnetic Bio-Plex assay (23-plex panel, cod. #M60009RDPD, Bio-Rad Laboratories, Hercules, CA) including the following molecules: Interleukin (IL)-1α, IL-1β, IL-2, IL-3, IL-4, IL-5, IL-6, IL-9, IL-10, IL-12(p70), IL-12(p40), IL-13, IL-17A, tumor necrosis factor (TNF)-α, IFN-γ, Macrophage Inflammatory Protein (MIP)-1α, MIP-1β, Eotaxin, Monocyte Chemoattractant (MCP)-1/(CCL2), Granulocyte Colony stimulating factor (G-CSF), Granulocyte Monocyte Colony Stimulating factor GM-CSF, Regulated on Activation-Normal T cell Expressed and Secreted (RANTES or CCL5), and KC. The analysis was carried out using 15 μl of plasma samples or conditioned media diluted according to the manufacturer’s instructions. Quantification was carried out on a Bio-Plex® 200 System (Bio-Rad) equipped with a magnetic workstation and a Bio-Plex Manager Software version 6.1. Results were expressed as pg/ml as previously reported [37].

### Analysis of niacin receptors expression

Niacin receptors HACR1, HCAR2 and HCAR3 were investigated analyzing expression data available from two public databases, namely GEPIA2 database [43] and GEO database [44]. The dimensionality reduction tool available on GEPIA2, at http://gepia2.cancer-pku.cn/#dimension, was exploited to perform the Principal Component Analysis (PCA) to show the genetic distance of melanoma to healthy controls according to the principal variance components of these three receptors. HCAR3 expression was also investigated on the GEO database [44] in two datasets investigating expression in normal skin vs melanoma samples (GDS1375, [45]) and in primary melanoma vs metastatic melanoma (GDS3966, [46]).

### Statistical analysis

Data are expressed as mean ± S.D. (unless differently specified) from at least three independent experiments. For in vitro studies, statistical analysis was performed using Student’s t-test or one-way ANOVA test when indicated. When needed, normal distribution was calculated by D’Agostino-Pearson omnibus normality test. *P* value ≤0.01 was considered the statistically significant threshold, unless differently specified.

SIRT2 activity data were analyzed with GraphPad Prism 5 (GraphPad Software Inc.) software as follows: upon log-transformation of the concentration data, nonlinear regression was carried out, choosing standard curve, dose-response stimulation, log (agonist) vs response and EC50 value calculation was carried out. For in vivo experiments Mann–Whitney U test for independent samples was used to evaluate the statistical significance of the difference between groups. Mouse survival was analyzed by the Kaplan-Meier method. The survival distributions of treatment groups were compared with the log-rank test. Differences in *p* values of 0.05 or less were considered significant. Statistical analyses were performed on GraphPad Prism 5 (GraphPad Software Inc.) and MedCalc software (MedCalc Software Ltd).

## Results

### Effect of NAM on human melanoma cells number

**Fig. 1:**
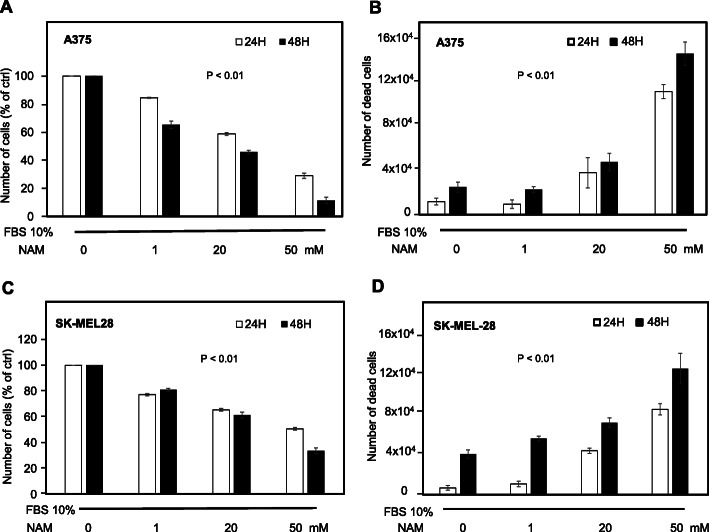
Cell number and cell death of human melanoma cells treated with increasing NAM concentrations. **a**, **c**: A375 and SK-MEL-28 cells were counted at 24 and 48 h following NAM treatment (1, 20, 50 mM). A375 (8 × 10^4^) and SK-MEL-28 (1 × 10^5^) cells were plated in p35 Petri dishes at time 0. Data are expressed as percentage of untreated cells. **b**, **d**: Cell death was quantified according to trypan blue exclusion assay and expressed as absolute numbers. All data are expressed as mean ± S.D. of three independent experiments. In each panel, statistical difference was achieved by one-way ANOVA test

**Fig. 2:**
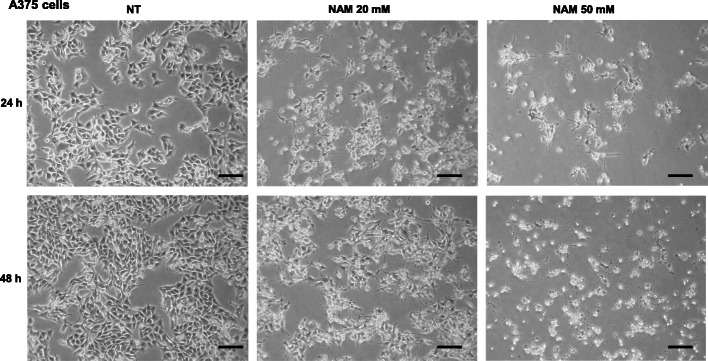
Representative images of A375. Representative fields showing A375 untreated (NT) or treated with NAM 20 and 50 mM for 24 and 48 h. (100x magnification). Scale bar = 50 μM

A375 and SK-MEL-28 cells were treated with increasing NAM doses (1, 20, and 50 mM), in the presence of 10% FBS. The cell number was counted at 24 and 48 h. Figure 1a and c show that NAM significantly reduces cell number in a dose-dependent manner with a strong inhibitory effect at 20 mM and an almost complete effect at 50 mM concentration. Figure 2 shows a representative field of A375 treated with NAM 20 and 50 mM at 24 and 48 h. Supplementary Figure 1 depicts a representative field of SK-MEL-28.

Significant increase of cell death was also observed at 24 and 48 h in both A375 and SK-MEL-28 cells (Fig. 1b, d). These data indicate a strong dose- and time-dependent anti-melanoma effect on both human melanoma cells, in the presence of 10% FBS. The inhibitory effects at 48 h depicted in panel A and C are more evident in A375 cells, which are more aggressive as compared to SK-MEL-28 cells, according to the Melanoma AGgressiveness Score (MAGS) we recently published [37].

### Effect of NAM on cell cycle

**Fig. 3:**
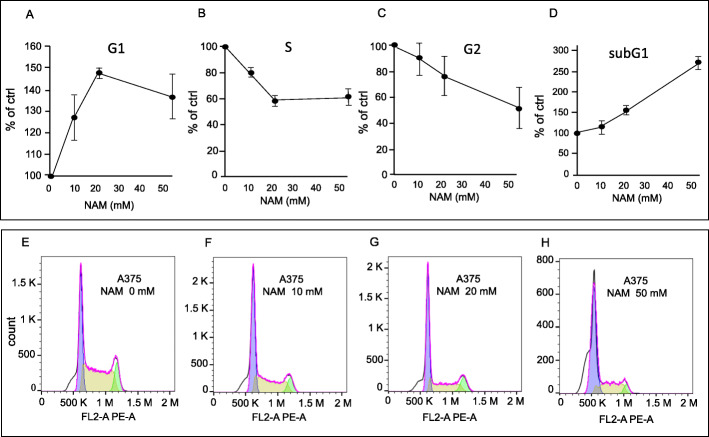
Cell cycle analysis of A375 cells treated with increasing doses of NAM. A375 cells were plated (8 × 10^4^ cells/plate) in p35 Petri dishes at time 0 and were then starved for 18 h in serum-free medium. Cells were then treated with 0, 10, 20, and 50 mM NAM, for 24 h, in the presence of FBS 10%. Panels **a**, **b**, **c**, **d** report the mean ± S.D. of three independent experiments; 10.000 events were acquired. Panels **e** to **h** show a representative experiment. One-way ANOVA test in panels **a**, **b** and **d** computed a *p* < 0.05

Cells were treated with increasing doses of NAM (10, 20, and 50 mM) and the effect on the cell cycle distribution was investigated at 24 h. Figure 3 shows that A375 cells undergo significant accumulation in G1 phase, significant reduction in S phase, and significant increase in the sub-G1 (apoptosis) phase (panels A, B, D respectively), according to one-way ANOVA test. Panels E to H show a representative experiment at each dose.

### NAD^+^, ATP and ROS levels in A375 and SK-MEL-28 following short-term NAM treatment

**Fig. 4:**
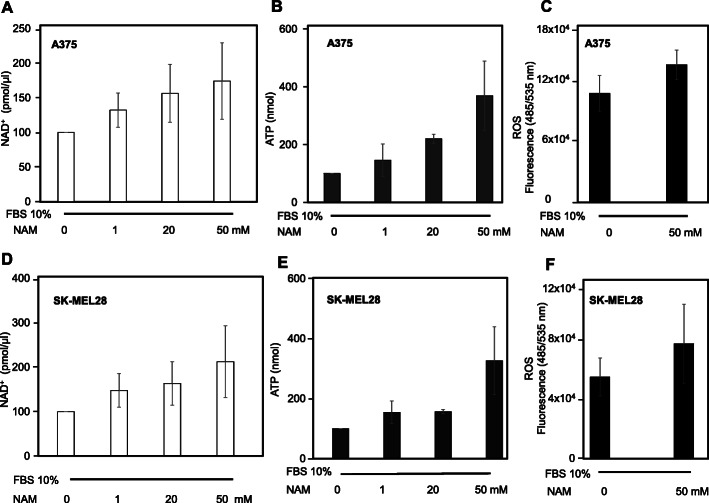
NAD^+^, ATP, and ROS levels under NAM treatment. **a**, **b**, **d**, **e**: NAM treatment increased NAD^+^ and ATP levels in both A375 and SK-MEL-28 cells (8 × 10^4^ and 1 × 10^5^ in p35 Petri dishes, respectively) after 6 h treatment. **c, f**: NAM increased ROS levels in A375 and SK-MEL-28 cells. The cells (8 × 10^4^ and 1 × 10^5^ in p35 Petri dishes, respectively) were stained with 2′,7′-dichlorodihydroflurescein diacetate (DCFDA) and then treated with 50 mM NAM for 6 h. All data are expressed as mean ± S.D. of three independent experiments. In panels **a**, **b**, **d**, **e** one-way ANOVA test as statistical assay computed a *p* < 0.05. In panels **c** and **f** a *p* < 0.05 was computed, according to Student’s t-test

NAD^+^, ATP, and ROS levels were measured in A375 and SK-MEL-28 cells. Figure 4a and d show that NAD^+^ is significantly increased upon 6 h treatment (at 1, 20, and 50 mM) in both cell types in a dose-dependent manner. Figure 4b and e show a parallel increase of intracellular ATP at 6 h. Finally, Fig. 4c and f show a significant increase of ROS levels in both cell types, upon 6 h treatment with 50 mM NAM. These data indicate that NAM exerts early strong metabolic effects.

### NAM effects on mouse B16-F10 cell number, cell death, and ROS levels

**Fig. 5:**
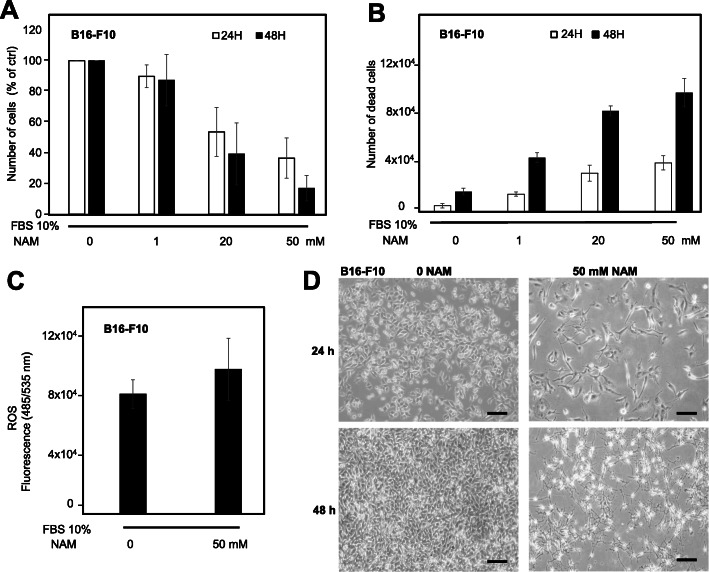
NAM effects on mouse B16-F10 melanoma cells. **a**: B16-F10 cells were counted after NAM treatment for 24 and 48 h. B16-F10 cells (8 × 10^4^) were plated in p35 Petri dishes at time 0, in the presence of 10% FBS. Cell number is significantly reduced in a dose-dependent manner. Data are expressed as percentage of untreated cells. **b**: Quantification of cell death by trypan blue exclusion assay, expressed as absolute numbers. **c**: ROS levels evaluated by 2′,7′- dichlorodihydrofluorescin diacetate (DCFDA) staining in cells treated with 50 mM NAM for 6 h. **d**: Representative fields of B16-F10 cells treated with NAM 50 mM for 24 and 48 h are shown (100x magnification). Scale bar = 50 μm. All data are expressed as mean ± S.D. of three independent experiments. In panels **a** and **b** one-way ANOVA test computed a *p* < 0.05. In panel **c** Student’s t-test computed a *p* < 0.05

We then investigated the in vitro effects of NAM in a mouse melanoma cell line. Figure 5a and b show a significant and time-dependent cell growth reduction and cell-death increase of B16-F10 mouse melanoma cells after NAM treatment (1, 20, and 50 mM); Fig. 5c shows a significant increase of ROS levels after 6 h treatment with NAM 50 mM. Figure 5d shows a representative field of B16-F10 cells treated with NAM 50 mM at 24 and 48 h. These data indicate that NAM anti-melanoma effects observed in human cells are confirmed in mouse melanoma cells.

### Sirtuin 2 (SIRT2) activity in vitro upon NAM treatment; SIRT2 transcriptomic analysis in silico

**Fig. 6:**
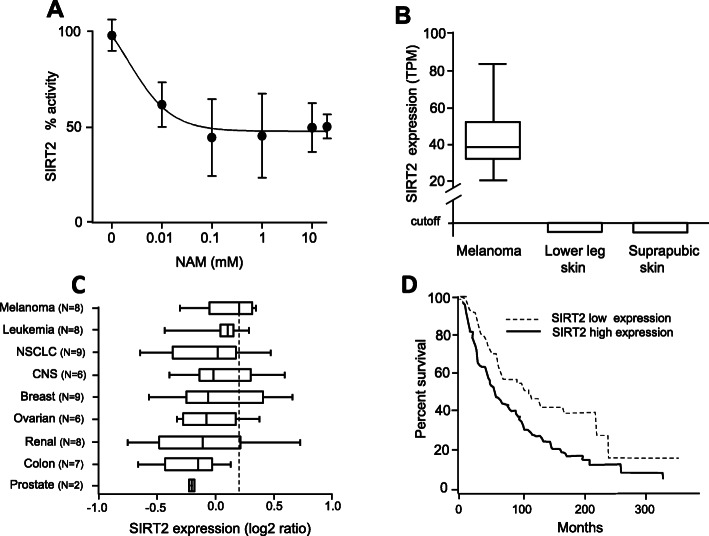
SIRT2 transcription in silico analysis and in vitro activity assay. **a.** SIRT2 in vitro activity upon NAM treatment. The activity was measured with the SIRT2 Inhibitor Screening Assay Kit (Fluorometric) (BioVision), as reported in Methods section. After 1 h incubation at 37 °C, the in vitro activity was found to be reduced by NAM in a dose-dependent manner. **b:** Expression level of SIRT2 in melanoma vs normal skin, according to “Pan-Cancer Analysis of Whole Genomes” reported by the Expression Atlas at EBI [42] (https://www.ebi.ac.uk/gxa/home). SIRT2 expression is strongly increased in melanoma as compared to control skin. **c.** The expression level of SIRT2 in NCI-60 human cancer cells lines, from GEO dataset GDS1761. Melanoma shows the highest median expression of SIRT2, according to the expression values in 8 human cell lines (namely LOXIMVI, M14, MALME-3 M, SK-MEL-28, SK-MEL-2, SK-MEL-5, UACC257, UACC62). Box & whisker plot reports median and 5–95 percentile. **d.** Overall survival in melanoma patients as a function of SIRT2 expression, according to expression data reported in GEPIA2 database; overall survival is computed from quartile distribution (the cutoff for high expression was set to 70%; cutoff for low expression set to 25%). Significant improvement of overall survival is observed in patients expressing low-levels of SIRT2 (HR = 1.9, *p =* 0.0004)

NAM effect on purified SIRT2 was then evaluated by an in vitro activity assay. Panel A of Fig. 6 shows that increasing NAM concentrations ranging from 0.01 mM up to 20 mM lead to a dose-dependent inhibition of SIRT2 enzymatic in vitro activity after 1 h incubation time. The computed EC50 was 2 μM. These data led us to hypothesize that inhibition of SIRT2 by NAM may underly, at least in part, the observed anti-melanoma effects. Sirtuins are indicated as possible therapeutic targets in different cancer types. According to expression values reported in GENT2 database, we report here for the first time that SIRT2 expression is significantly increased (*p* < 0.0002) in metastatic melanoma patients (expression value 8.3 + 0.43 in 148 patients) as compared to controls (expression value 8.09 + 0.59, in 211 healthy subjects). This finding was validated in a different database, namely the “Pan-Cancer Analysis of Whole Genomes” reported by the Expression Atlas at EBI [42]. Panel B of Fig. 6 shows that SIRT2 expression in 36 melanoma samples is strongly increased as compared to sun-exposed and sun-non exposed normal skin. Furthermore, SIRT2 shows the highest expression value in 8 melanoma cell lines (including SK-MEL-28) as compared to several other cancer lines (Panel C of Fig. 6). Furthermore, SIRT2 expression level significantly relates to an improved overall survival while the expression of other sirtuins shows no significant relation (see Table 1). Overall patients’ survival is depicted in Panel D of Fig. 6: patients with higher expression show lower survival with a strong and significant Hazard Ratio for patients with high expression (HR = 1.9, *p* = 0.0004). Altogether these data indicate that SIRT2 expression is increased in melanoma and patients with lower expression show improved survival.

**Table 1:** Relation of SIRT1, SIRT2, SIRT3, SIRT4, SIRT5, SIRT6, and SIRT7 expression with overall survival Hazard Ratio in melanoma patients, according to data reported in GEPIA2 database [43]. SIRT2 shows strong and significant HR

Gene name	Hazard Ratio for high expression	*P*	N. of patients with high expression	N. of patients with low expression
SIRT1	0.72	0.074	138	114
SIRT2	1.9	0.0004	138	115
SIRT3	0.75	0.12	138	114
SIRT4	0.91	0.6	137	109
SIRT5	0.98	0.93	138	115
SIRT6	0.98	0.93	138	115
SIRT7	0.91	0.63	138	115

### In vivo NAM antitumor activity in a murine metastatic melanoma model

**Fig. 7:**
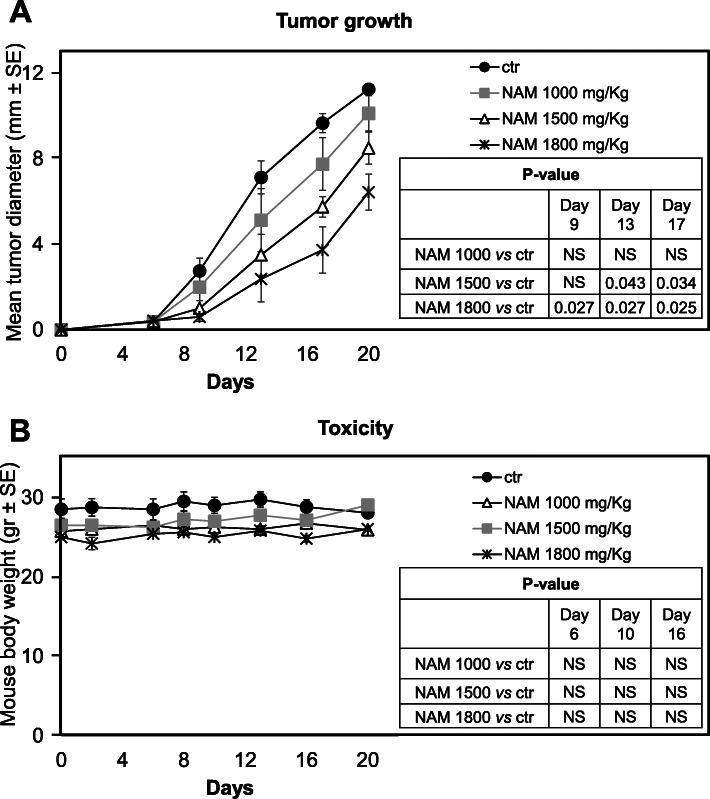
Antitumor effect and toxicity of NAM administered 5 days per week, i.p. C57BL/6 mice were injected s.c. with 3 × 10^5^ B16-F10 cells. Mice were divided into four groups (*n* = 4) and treated i.p. with NAM 1000 (grey square), 1500 (open triangle) or 1800 (asterisk) mg/Kg or with saline (ctr, black circle), as indicated, starting from the same day of tumor implant for 5 days per week. **a**: Tumor growth was monitored twice a week with a digital caliper. Data are reported as the average of the mean tumor diameter of 4 mice per group ± S.E. **b**: Mouse body weight was monitored twice a week as an indication of NAM toxicity. Data are reported as mean weight of 4 mice per group ± S.E. *P*-values were calculated by Mann-Whitney U test for independent samples (NS, not significant)

**Fig. 8:**
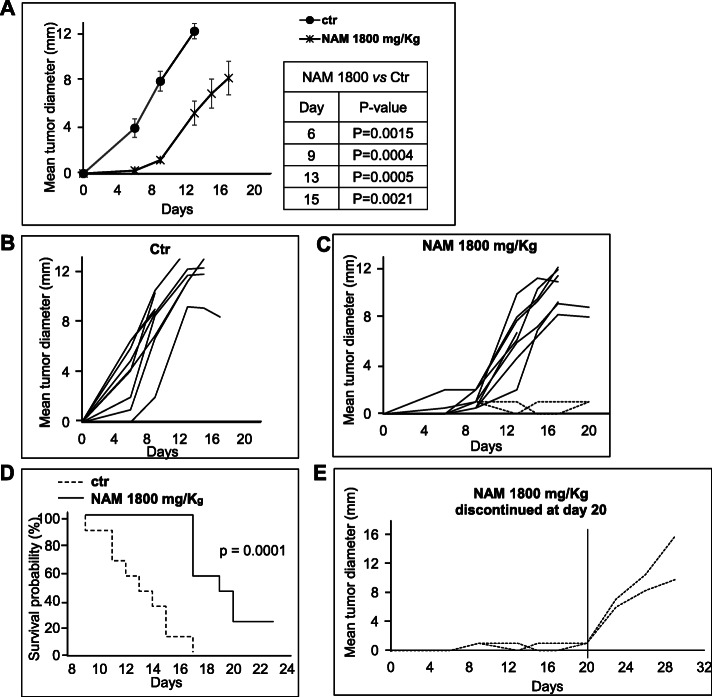
Antitumor efficacy of NAM 1800 mg/Kg administered every day, i.p. C57BL/6 mice were injected s.c. with 3 × 10^5^ B16-F10 cells. Mice were divided in two groups and treated i.p. with NAM 1800 mg/Kg (*n* = 10) or with saline (ctr) (*n* = 9), as indicated, starting from the same day of tumor implant for 7 days per week. Tumor growth was monitored twice a week with a digital caliper. P-values were calculated by Mann-Whitney U test for independent samples. **a**: Data are reported as the average of the mean tumor diameter of 10 or 9 mice per group ± S.E. **b, c**: Data are reported as mean tumor diameter of each individual mouse. **d**: Kaplan-Meier survival plots of saline- (dashed line) or NAM-treated (solid line) tumor-bearing mice. The endpoint used for survival analyses was the day when the tumors reached a mean diameter ≥ 12 mm or the day of euthanasia (mice with necrotic tumor, weight loss, or sign of distress). P-values were calculated using the log-rank test. **e**: Tumor growth of two NAM-treated mice (same as in C) after treatment discontinuation at day 20

B16-F10 melanoma cells (3 × 10^5^) were transplanted into C57BL/6 mice. Dose-response experiments were carried out to evaluate the antitumor efficacy and the toxicity of three NAM doses administered systemically (i.p.). Sixteen mice, divided into four groups (4 mice per group), were treated i.p. with NAM 1000, 1500 and 1800 mg/Kg or with saline as a control, for 5 days per week, followed by 2-day rest. Figure 7a shows tumor growth curves in the 4 groups. At 1500 and 1800 mg/Kg, NAM significantly delayed tumor growth (Fig. 7a). Mouse body weight was monitored twice a week as an indication of NAM toxicity. Mice were also inspected for signs of distress. Figure 7b shows that NAM administration did not alter significantly the mouse body weight. Nevertheless, all mice were sleepy for a few hours after treatment regardless of the dose used. An additional set of experiments was carried out: nineteen mice were treated i.p. seven days per week with 1800 mg/Kg of NAM (*n* = 10) or saline as control (*n* = 9). Figure 8a shows the mean tumor growth curves of saline- vs NAM-treated mice. A significant (*p* < 0.01) delay of tumor growth was observed in NAM-treated mice at all time points. Panels B and C of Fig. 8 show the growth curves of saline-treated and NAM-treated individual mice, respectively. While in control mice the mean tumor diameters ranged between 1 and 6.5 mm 6 days following tumor implant and between 2 and 10 mm at day 9 (Fig. 8b), the tumors were palpable only in two NAM-treated mice at day 6 and were all ≤2 mm at day 9 (Fig. 8c). Following day 9, tumors grew in 8 out of 10 NAM-treated mice even if the difference in size with saline-treated mice was still significant. Of special note, in 2 NAM-treated mice the tumor diameter was only 1 mm up to 20 days following tumor implant, indicating a long-lasting antitumor effect (Fig. 8c). Interestingly, in these two mice treatment discontinuation led to tumor regrowth (Fig. 8e). Figure 8d shows the Kaplan-Meier survival plots in saline- vs NAM-treated tumor-bearing mice. The endpoint used for survival analyses was the day when the tumors reached a mean diameter ≥ 12 mm or the day of euthanasia (mice with necrotic tumor, weight loss, or sign of distress). The survival curve of NAM-treated mice was significantly different from the curve of control mice. Starting from day 9 to day 17 following tumor implant all saline-treated mice reached the endpoint. On the contrary, NAM-treated mice reached the endpoint starting from day 17 and 20% of mice did not reach the endpoint while on treatment. The toxicity of the seven-days-per-week administration schedule was like that of the 5-days-per-week schedule (data not shown). Overall, these data indicate that NAM treatment induces a significant, dose-dependent delay of tumor growth in a metastatic tumor model; 1800 mg/Kg treatment also induces a significant increase in the survival of tumor-bearing mice.

### In vivo immunostimulating effects of NAM treatment

**Fig. 9:**
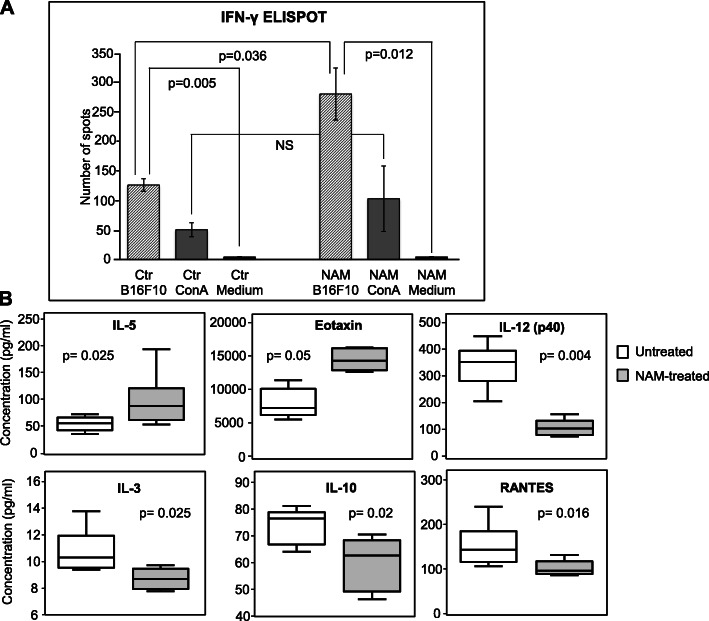
NAM treatment induces an anti-tumor immune response and cytokine/chemokine modulation. C57BL/6 mice were transplanted with B16-F10 tumor cells and treated every day with NAM (1800 mg/Kg) as in Fig. 8. **a:** The frequency of interferon (IFN)-γ producing cells in the blood of mice treated with saline (ctr) or with NAM was analyzed 14 days after tumor implant. Blood was collected by individual mice (*n* = 6) and IFN-γ ELISPOT assay was conducted to evaluate the response against irradiated (20 Gy) B16-F10 cells. Medium or concanavalin A (ConA) (5 μg/ml) were used as negative and positive control, respectively. Data represent the mean (± S.E.) of 6 individual mice tested in triplicate. P-values were calculated by Mann-Whitney U test for independent samples. **b:** NAM treatment significantly modulates the protein levels of cytokines and chemokines in the plasma of tumor-bearing mice. The protein level of 23 cytokines, chemokines, and growth factors was analyzed by Bio-Plex cytokine assay in saline- (white boxplot) or NAM-treated (grey boxplot) mice. The boxplots contain median values (horizontal line), interquartile range (the box length), highest and smallest values (whiskers) of the plasma concentration (pg/ml) of the indicated cytokine or chemokine measured in 6 individual mice per group. *P* values were calculated by Mann-Whitney U test for independent samples

Since NAM administration is known to reduce UV-mediated immunosuppression [47], we explored whether NAM affected some components of the anti-tumor immune response. We compared the frequency of IFN-γ producing cells in the blood of NAM-treated (1800 mg/Kg) and saline-treated (ctr) tumor-bearing mice (Fig. 9a). The mice were the same of the experiments depicted in Fig. 8. Fourteen days following the tumor implant (i.e. at a time point when the tumor growth curves were significantly different), blood was collected by individual mice and IFN-γ ELISPOT assay was conducted to evaluate the response against irradiated (20 Gy) B16-F10 cells. Medium or concanavalin A were used as negative and positive control, respectively. Figure 9a shows that in both saline- and NAM-treated mice a significant production of IFN-γ in response to irradiated melanoma tumor cells was observed, indicating the presence of a spontaneous anti-tumor immune response. Remarkably, in NAM-treated mice the frequency of IFN-γ producing cells was significantly higher than in control mice. These data indicate that NAM treatment affects a key mediator of cell-mediated anti-tumor immunity, such as IFN-γ.

Then, the plasma levels of 23 cytokines, chemokines, and growth factors were measured in NAM- vs saline-treated mice. Plasma was derived from the same blood used for the ELISPOT assay samples (14 days following tumor implant). Figure 9b shows that NAM treatment significantly modulates the protein levels of 4 cytokines and 2 chemokines in the plasma of tumor-bearing mice, namely, IL-3, IL-5, IL-10, IL-12(p40), Eotaxin and RANTES. Eotaxin and IL-5 were significantly increased in NAM-treated mice, whereas IL-3, IL-12(p40), RANTES and IL-10 protein levels were significantly reduced.

Increased levels of IL-5 and Eotaxin may suggest an effect of NAM on CD4^+^ Th2 cells and on eosinophils [48]. Altogether these data show that NAM treatment of tumor-bearing mice induces immunomodulation affecting different immune subsets.

### Expression levels of niacin receptors in human melanoma specimens

**Fig. 10:**
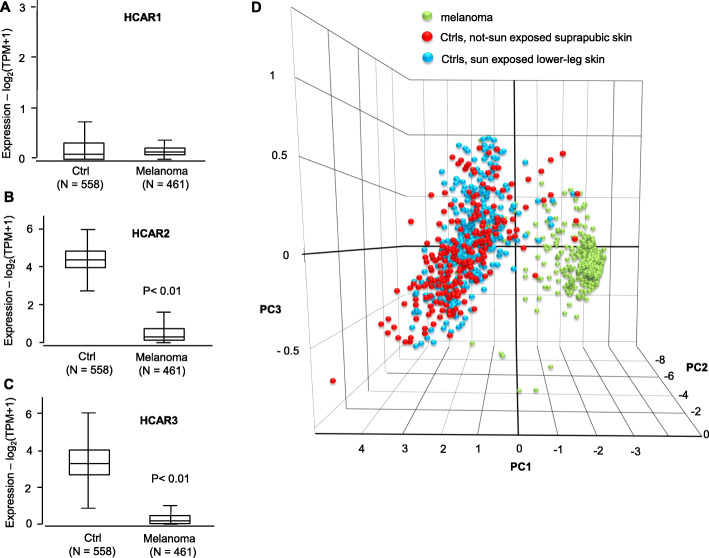
Expression level of Niacin receptors HCAR2 and HCAR3. **a**, **b**, and **c**: expression levels of the Niacin receptors HCAR2 and HACR3, and of the other from HCAR1, in 558 controls and 461 melanoma samples from GEPIA2 database. All three forms have very low expression levels, significantly different from controls for HCAR2 and HCAR3, i.e. the high- and low- affinity receptors of Nicotinic acid respectively. **d**: PCA analysis of the combined values of HCAR1, HCAR2 and HCAR3 carried out at the link: http://gepia2.cancer-pku.cn/#dimension. Melanoma samples appear clearly separated from controls in the PCA-made three-dimensional space. *P* values were calculated by t-Student test

NAM is normally acquired via the daily diet; we hypothesized that the daily protecting action of NAM may be reduced or absent in melanoma patients. NAM deamidation produces Nicotinic acid which acts via the high- and low- affinity receptors HCAR2 and HCAR3. We analyzed the expression levels of HCAR2 and HCAR3 in GEPIA2 database and on the independent GEO database, in a total of 1172 patients. GEPIA2 analysis indicated a strong reduction of HCAR2 and HCAR3 expression in melanoma patients as compared to normal skin controls. HCAR1 is an additional receptor isoform and has low expression levels in both normal skin and in melanoma samples (Fig. 10a, b, c). Furthermore, their expression levels were analyzed by PCA (Principal Component Analysis). Panel D of Fig. 10 shows the three-dimensional space defined by the 3 variance components associated with the expression values of these receptors. When observed in this space, a striking separation between melanoma and healthy controls is evident.

Expression data of Niacin receptors were then investigated in the independent database GEO [44] (available at https://www.ncbi.nlm.nih.gov/gds). It only reports the expression values of HCAR3. Supplemental Figure 2 shows a strong and significant reduction of the expression levels in melanoma samples as compared to control skin (from dataset GDS1375), and a strong and significant reduction in metastatic melanoma samples as compared to primary melanoma samples (from dataset GDS3966), therefore confirming the expression data observed in GEPIA2 database.

These data indicate that Niacin receptors expression is strongly reduced in melanoma patients suggesting that the physiological protective action of NAM may be reduced in melanoma patients due, at least in part, to the strong reduction of these receptors.

## Discussion

Advanced melanoma patients have different therapeutic options including immunotherapy and targeted therapy. However, the onset of resistance and of severe side effects limit the percentage of patients with long-lasting responses. It is therefore urgent to identify new effective approaches [49]. NAM has beneficial therapeutic effects on joints, pancreatic beta cells, kidney and skin. It reduces acne severity and reduces the incidence of many types of non-melanoma skin cancers and keratoses [50]. NAM shows different, even opposite, effects at low vs high doses. In fact, at doses near to 5 mM NAM shows cell protection activity improving viability and replication potential of cells in culture, while at doses above 20 mM, it causes apoptotic death, with an IC50 of 21.5 mM [23]. In the present study, NAM showed a strong and significant antiproliferation effect on A375 and SK-MEL-28 melanoma cells, two human melanoma cell lines characterized for high and low aggressiveness, respectively [37]. NAM was tested in the mM concentration range since similar high concentrations have recently shown activity in melanoma cells [23] and in colon cancer cells [51]. In the latter cells low- and high-doses of NAM show opposite effects; micromolar doses appear to be cell-protective while millimolar doses induce cell death, likely related to the oxidative stress [51]. Cell killing effects of high NAM doses were also observed in *C. elegans* with defective activity of nicotinamidase PNC-1, where NAM levels increase tenfold [52].

NAM is known to play an important role in energy production and metabolism [53] and is reported to increase NAD^+^ and ATP levels in skin cells [12, 53]. Data reported in the present study support a mechanism likely related to an early alteration of cellular energy metabolism. In fact, we observed that NAM treatment increases NAD^+^, ATP, and ROS levels at as early as 6 h treatment and leads to a significant increase of cells in G1 phase and relevant depletion of cells in S-G2 phase and a strong increase of cells in apoptosis (sub-G1 phase) at 24 h. Of note, NAM has been shown to induce apoptosis also in mouse teratocarcinoma stem cells [54] and increased intracellular levels of ATP have been related to cytotoxic effects in other cell types [55]. All such early effects paralleled the relevant cells number reduction and cell death induction observed at 24 and 48 h treatment.

ROS are important pathophysiological molecules involved in vital cellular processes. Excessive ROS levels can result in oxidative stress ultimately leading to cell death. ROS homeostasis is often imbalanced in cancer and an elevated oxidative status has been associated with melanoma. In cancer cells, ROS may induce and maintain the oncogenic phenotype and, on the other hand, induce cellular senescence and apoptosis [56]. In this regard, elevated ROS production induced by a chalcone derivative has been shown to significantly reduce melanoma cells viability [57]. Similarly, ROS generation was shown to mediate a proapoptotic activity on human melanoma cells by different molecules, namely chaetocin, a small molecule of fungal origin, and oxalomalate [58, 59]. Data presented in the current study suggest that NAM, via an early effect, directly interferes with melanoma cells metabolism and oxidative stress, indicating a potent anti-melanoma effect besides the well-known ability to reduce non-melanoma skin cancer incidence [11]. In the current study most of the effects observed in human cells were confirmed in mouse melanoma cells B16-F10, namely, reduction of cell growth, induction of cells death and increase of ROS levels.

Sirtuins are interesting molecular targets in melanoma. In mammals, seven homologs are known, namely SIRT1 to SIRT7, ubiquitously expressed and involved in many biological functions such as the control of gene expression, cell cycle, apoptosis, DNA repair, metabolism, and aging. SIRT2 is a cytoplasmic and nuclear protein. While it is downregulated in many cancers such as breast cancer, prostate cancer, human gliomas, neck squamous cell carcinoma, colorectal cancer and leukemia [60–65], we report here, according to other studies [66], that SIRT2 is upregulated in melanoma. In fact, it is reported both as tumor promoter and as tumor suppressor [67]. Karwaciak and colleagues [68] demonstrated that the SIRT2 inhibitor AC-93253 inhibits the expression of genes involved in the progression and chemoresistance of melanoma, influencing proliferation and apoptosis. They also demonstrated that SIRT2 inhibition makes melanoma cells sensible to dasatinib [69]. Furthermore, SIRT1&2 knockdown inhibits proliferation and decreases colony formation in melanoma cells [66]. We also report here for the first time a possible role of SIRT2 expression levels on the overall survival of melanoma patients. The EC50 of NAM on SIRT2 activity in vitro was measured here at 2 μM, suggesting that NAM anti-melanoma activity may be related, at least in part, to the SIRT2 inhibition. As recently reviewed [65], nicotinamide phosphoribosyltransferase (NAMPT) plays a critical role in NAD^+^ synthesis and energy control and its role in melanoma, mediated by BRAF and Sirtuins, is being recognized with increasing evidence. In the present study NAM is reported as a strong SIRT2 inhibitor with an EC50 value, comparable to other inhibitors such as Sirtuin-rearranging ligand (SirReal2) and Thiomyristoyl [70, 71]. NAM doses between 5 and 20 mM are known to induce mitophagy with a mechanism likely related to the NAD^+^ increase [72]; apoptosis and mitophagy were shown to be SIRT2 dependent in the highly aggressive MDA-MB-231 breast cancer cells [73]. We therefore hypothesize that a SIRT2-dependent mitophagy process may occur and further investigation is ongoing in this regard.

The NAM strong effects observed in vitro were paralleled by the relevant effects achieved in vivo. Systemic treatment of melanoma-bearing mice with NAM (1500–1800 mg/Kg) significantly inhibited melanoma growth in vivo in a mouse transplanted melanoma model. Previous reports have shown that NAM at low dose (30 mg/kg i.p.) is protective in various chemical- and ultraviolet radiation (UVR)-induced carcinogenesis in animal models (reviewed in Reference [66]. On the other hand, at high doses (1000 mg/kg i.p.) NAM inhibits the growth of transplanted murine breast adenocarcinoma and carcinogen-induced liver tumors in mice (reviewed in [74]). Here we show a significant dose-dependent anti-melanoma effect by daily treatment of tumor-bearing mice with high NAM doses (1500–1800 mg/Kg i.p.), no severe toxicity (see Fig. 7b). In addition to delaying tumor growth, 1800 mg/Kg NAM significantly increased mice survival. Since the reported LD50 in mice is 2500 mg/Kg i.v [67]., cautions will be needed to establish the maximum tolerated dose (MTD) in humans due to toxicity risk. By using the body surface area method [75], the mouse dose of 1800 mg/Kg corresponds to 146 mg/Kg in humans, i.e. 8.76 g/day for a 60 kg person. Although further studies are needed to establish the MTD and the biologically active dose (BAD) in melanoma patients, we do not anticipate severe toxicity. In fact, NAM administration up to 3 g daily is well tolerated [76]. Mild and transient side effects (such as headache, dizziness, and vomiting) were reported in healthy humans with doses up to 6 g [77]. Reversible hepatoxicity was observed with 9 g/day (76). It should be noted also that NAM under our experimental conditions needs to be administered at the beginning of the tumor onset and the treatment needs to be continuous to prevent the tumor regrowth.

Immunohistochemical analysis of melanomas arising in NAM- or placebo-treated patients within the ONTRAC skin cancer chemoprevention trial [11] demonstrated that melanoma lesions occurring in NAM-treated patients were more infiltrated by CD4^+^ and CD8^+^ lymphocytes than in the placebo, indicating a contribution of the immune response to its pharmacological effect [23].

Since IFN-γ is critical for T cell-mediated tumor regression [78], we analyzed the effect of NAM treatment on the frequency of IFN-γ-secreting cells in response to melanoma cells in PBMCs of tumor-bearing mice. IFN-γ-mediated anti-tumor response was strongly enhanced by NAM. NAM treatment also a significantly reduced the plasmatic levels of IL-3, IL-10, IL-12 and RANTES, and significantly increased IL-5 and Eotaxin levels, suggesting that NAM may trigger a complex modification of the cytokine/chemokine balance. Eotaxin is a potent chemoattractant for eosinophils toward inflammation sites in response to parasitic infections as well as in allergic and autoimmune diseases. Eotaxin binds CCR3 receptor expressed on eosinophils, basophil and Th2 lymphocytes, characterized by the release of Th2 cytokines such as IL-4, IL-5, IL-13, therefore Eotaxin plays a central role in mediating immune response toward a type-2 (Th2) profile [79].

Interestingly, the Th2 cytokine IL-5 was significantly increased in the plasma of NAM-treated melanoma-bearing mice, while IL-12 (p40), a typical Th1 cytokine, was decreased, indicating a shift toward a Th2 profile in NAM-treated melanoma-bearing mice. Although Th2-mediated immunity has traditionally been viewed as favoring tumor growth, the Th2 immune response was related to an anti-tumor activity especially if associated with eosinophils activation and release of eosinophil-associated cytotoxic granules [80], and Th2 cells were proposed in anticancer adoptive immunotherapy protocols [81]. Remarkably, eosinophilia is emerging as an important biomarker associated with prolonged survival of melanoma patients independently of therapy and in patients treated with checkpoint inhibitors [82].

NAM-treated mice also showed significant reduction of RANTES, IL-10 and IL-3 levels (Fig. 9). RANTES is a pro-inflammatory cytokine with a tumor-promoting role [83]; it is expressed in melanoma and is involved in controlling tumor growth and progression [84].

Furthermore, IL-10 has a known immunosuppressive effect in melanoma [85]. IL-3 is a multipotent hematopoietic growth factor produced by activated T cells, monocytes/macrophages and stroma cells, which was shown to promote tumor angiogenesis [86]. It is possible to speculate that its decreased levels may interfere with the tumor vasculature. Therefore, taken together, reduced levels of RANTES, IL-3 and IL-10 may be involved in the anti-melanoma immune response under NAM treatment.

NAM deamidation produces Nicotinic acid [87], which is known to bind Niacin receptors HCAR2 (or GPR109A or HM74A) and HCAR3 (or GPR109B or HM74B), respectively high- and low- affinity receptors [88]. Their activation has a known anti-inflammatory activity. To our knowledge, Niacin receptors have never been investigated in melanoma. We report here for the first time that expression of Niacin receptors HCAR2 and HCAR3 is almost completely lost in melanoma patients and the PCA (Principal Component Analysis) carried out on the combined expression of Niacin receptors revealed their ability to separate melanoma patients from healthy controls, indicating Niacin receptors as potentially relevant melanoma markers. Additional investigations are necessary on the role of NAM and Niacin receptors; however, according to these preliminary analyses, we argue that the biological effects of NAM and Nicotinic acid mediated by these receptors may play a key role in melanoma patients.

## Conclusion

The current study presents for the first time strong in vitro and in vivo anti-melanoma activity of NAM and several data indicating the underlying molecular mechanisms. NAM daily treatment has been previously shown to play a key role to prevent relapses of non-melanoma skin cancer; the present study gives a strong rationale for further investigations on the role NAM may play in melanoma treatment.

## Supplementary information

**Additional file 1: Supplementary Figure 1.**

**Additional file 2: Supplementary Figure 2.**

## Data Availability

n/a

## Abbreviations

NAM: Nicotinamide
ROS: Reactive Oxygen Species
DCFDA: 2′,7′-dichlorodihydroflurescein diacetate
SIRT1 to SIRT7: Sirtuin 1 to Sirtuin 7
IFN: Interferon
PCA: Principal Component Analysis
