# Supplementary material for: Nicotinamide inhibits melanoma in vitro and in vivo
Source: J Exp Clin Cancer Res. 2020 Oct 7;39:211. doi: 10.1186/s13046-020-01719-3 (PMC7542872; doi:10.1186/s13046-020-01719-3)
Supplement: Supplementary file 1 — Additional file 1: Supplementary Figure 1. [file 13046_2020_1719_MOESM1_ESM.pdf]

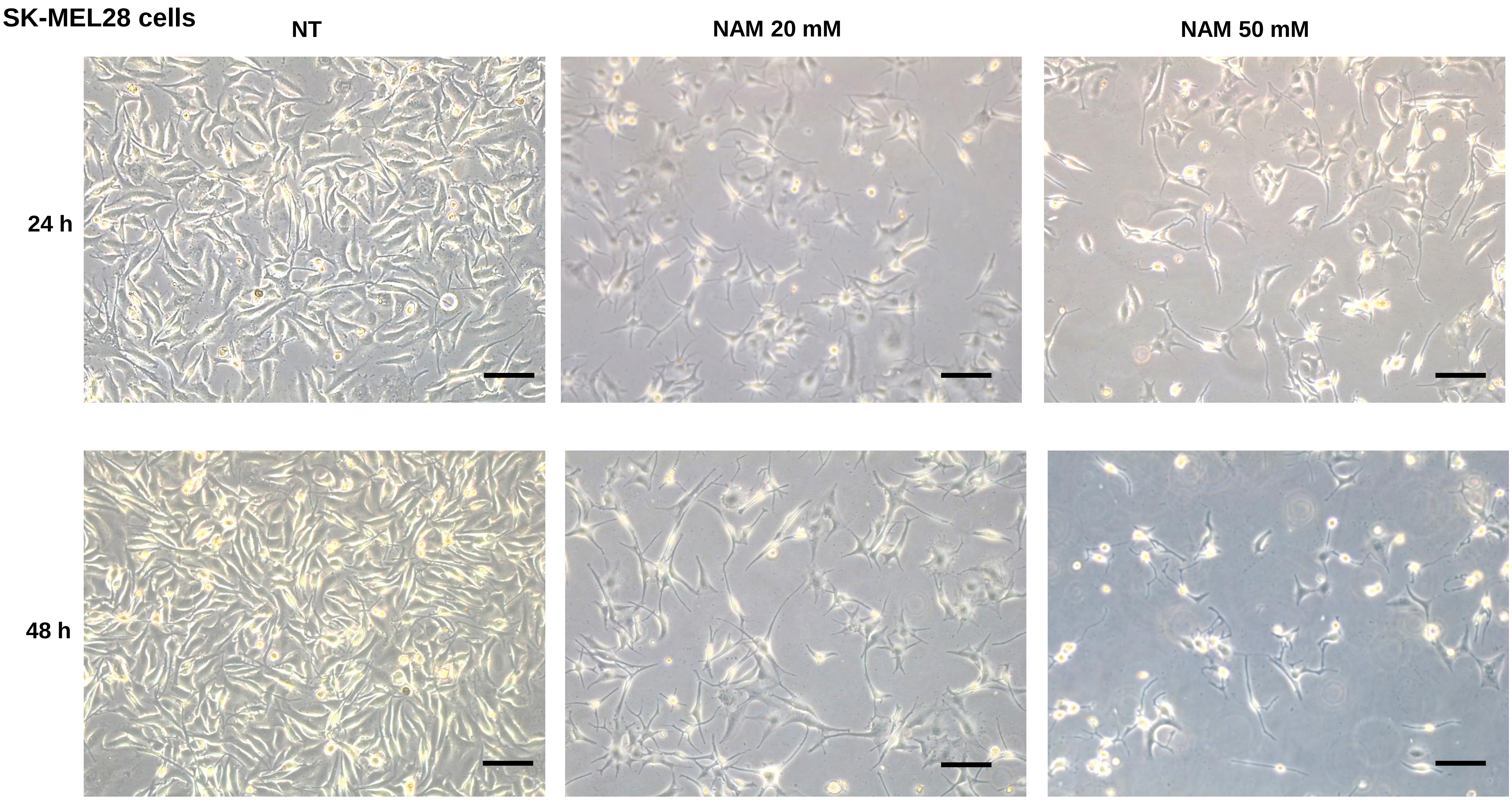

Supplementary Figure 2 Representative images of SK-MEL-28
Representative fields showing SK-MEL-28 untreated (NT) or treated with NAM 20 mM and 50 mM for 24 h and 48 h (100x magnification). Scale bar = 50 μm.
