# Supplementary material for: Nicotinamide inhibits melanoma in vitro and in vivo
Source: J Exp Clin Cancer Res. 2020 Oct 7;39:211. doi: 10.1186/s13046-020-01719-3 (PMC7542872; doi:10.1186/s13046-020-01719-3)
Supplement: Supplementary file 2 — Additional file 2: Supplementary Figure 2. [file 13046_2020_1719_MOESM2_ESM.pdf]

Figure 2 Sup

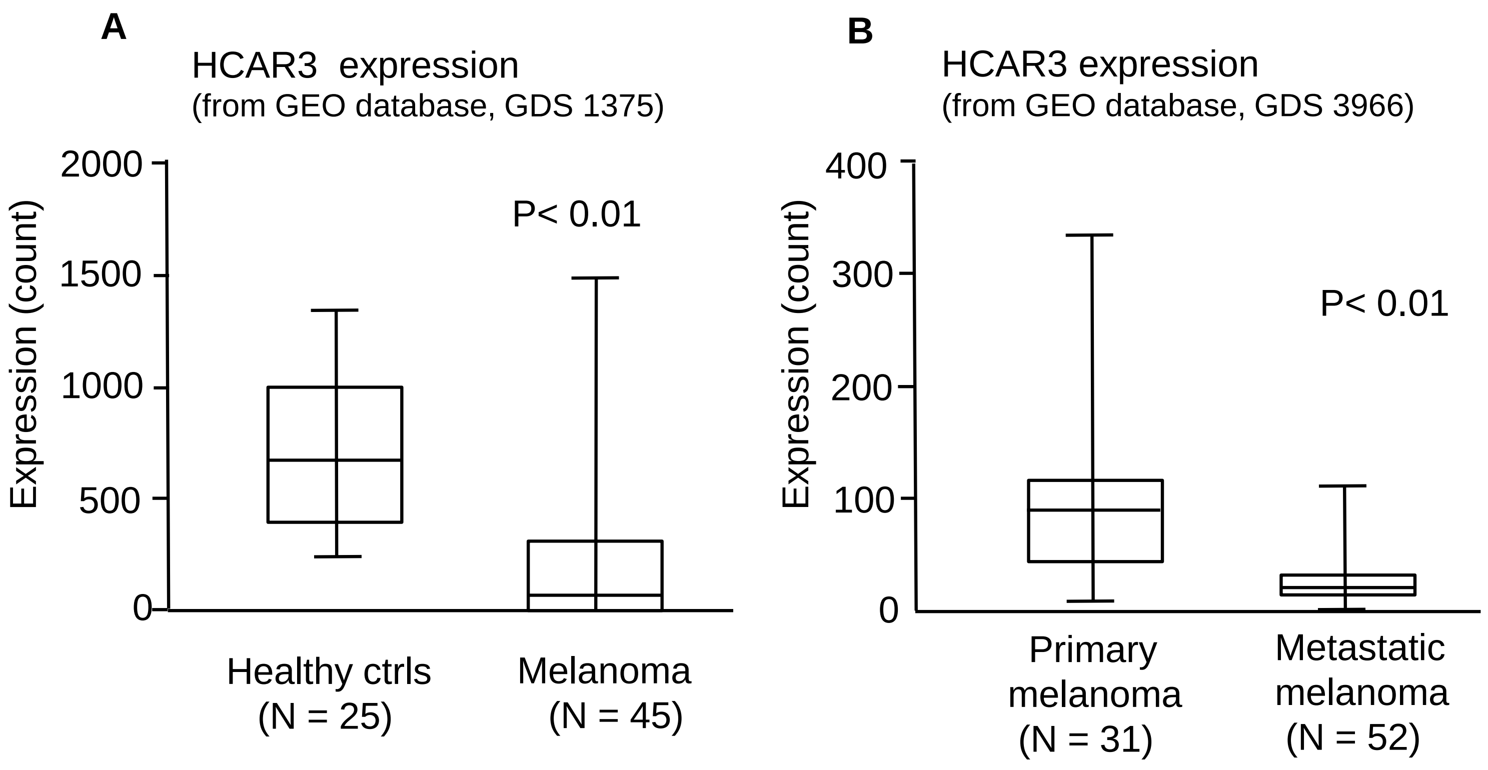

Expression of HCAR3 receptors according to datasets form GEO database. These data validate data from GEPIA2 database, indicating that expression of HCAR3 is strongly reduced in melanoma samples (GDS 1375)and almost abolished in metastatic vs primary melanoma (GDS 3966). HCAR2 receptors is not present in GEO datasets
